# Indents

**Published:** 1908-07-15

**Authors:** 


### INDENTS.

**☞ Brief Articles of Technical or Scientific Value will receive notice and comment. Contributions invited**

**Antiseptic Spray for Teeth and Gums.** In cleaning teeth I frequently follow the stick and ribbon floss with the brush wheel used upon the coronal surfaces. After this is all done, I use an antiseptic spray under a pressure of thirty pounds, and go all over the teeth, between them, and especially under the gum margin, to thoroughly wash out any debris that may be left. The spray I use is echefolta, 1 ounce; dioxygen, 5 ounces, and water, 4 ounces. —J. V. Conzetti in *Review.*

**A Varnish for Impressions.** Sodium silicate and ammonium hydroxide equal parts, forms a varnish that is indispensable for plaster molds, impressions, etc., and is inexpensive. For impressions—apply in the same manner as you would shellac. A coloring matter, as carmine, may be added so that the division line may be readily seen. Separate in the same manner as with shellac. When used on models in flask before packing the rubber the plaster readily separates from the plate after vulcanizing, leaving it clean and smooth.—T. A. Leach in *Review.*

**Canal Treatment.** I very seldom attempt to drill out and fill all the roots of the bicuspids and molars, but devitalize, open up the pulp chamber, then open the mouth of the root canals with Dr. J. Leon Williams' triangular treamers, wipe out the same with perhydrol ten per cent, then with a solution, absolute alcohol 1 oz., mercury bichloride 1 gr., and fill the mouths of the canals and pulp chamber with mummifying paste. The Soderbury formula with aristol or iodoform is used and the tooth sealed with the filling the case may require.—Dr. W. H. Jones in *Items.*

**To Make Wax Trial Plates Adhere to the Mouth.** All of the older practitioners have heard of powdered gum tragacanth, that some dentists give their patients when they insert the upper plate, telling them if it does not stay to sprinkle a little gum tragacanth on it, and it will stick. I am happy to say that I have not felt the need of that. There are times, however, when it is distinctly useful. When inserting a trial plate to get the bite or to see that the teeth are properly mounted so as to meet all requirements, by sprinkling the palatal surface with gum tragacanth any trouble from the plate not remaining in position will be obviated.—*Digest.*

**Objection to Immediate Root-Filling.** I do not believe that immediate filling of the root-canal should be attempted after removal of the pulp by pressure anesthesia, for the reason that the cocain not only anesthetizes the pulp, but a considerable area of the soft tissues at and adjacent to the apical foramen, so that, when filling the root there is no way of determining when the end has been reached by reason of these tissues being devoid of sensation. If the operator uses a method, and has the skill to fill roots to the end, he will find, if he fills immediately, that he is more than likely to carry the root-filling into the soft tissue beyond the apical foramen, thus causing the patient much discomfort, and perhaps, the loss of the tooth from continued irritation. —A. J. Cotterell, *Dental Brief.*

**Difficult Impressions.** One may occasionally have cases where it is difficult, if not impossible, to insert an ordinary impression tray sufficiently large to take the impression of the dental arches, owing chiefly to the smallness of the external opening. One can stretch the lips and angles of the mouth to their fullest extent without hurting the patient, if one takes a double piece of note paper about an inch and a half wide and three inches long and places that on the right side of the patient's mouth; then, as the tray is inserted sideways on the left side, the paper on the other side is made to act as a retractor, perfectly protecting the side of the mouth from abrasion and permitting the tray to slide in quite easily.—Harry Rose, *British Journal of Dental Science.*

**Opening into Abscessed Cavities.** Within the last few years I have modified my methods of operating on these cases considerably. Formerly I made an incision reaching to the bone, dissecting back the periosteum, opening into the cavity, curreting it out, cutting off the root, and packing it to force the parts away so that granulations might form and fill the cavity and thus bring about repair. I have adopted a different plan, which I know will serve my purpose better. Instead of holding away the overlying gums and overlying periosteum over the bone, I make a funnel-shaped opening, taking away this tissue and leaving a saucer-shaped cavity, and there is no packing required. Granulations will form, the cavity will fill, and repair will follow without any depression in the part.—Truman W. Brophy, in *Review.*

**Guthymol.** Thymol, added to gutta-percha, makes a very useful preparation for dental purposes. It possesses very desirable working properties, setting slowly and becoming hard. I have used it successfully as a temporary stopping and filling material and to crowd away overhanging gum margins and to obtain impressions of cavities. To obtain a satisfactory grade of guthymol add to base-plate gutta-percha a five per cent solution of thymol and soften the gutta-percha under heat. A mixture of guthymol, oil of cajuput, and a few fibres of asbestos makes an excellent root-canal filling. When ready to fill a cavity add to the required amount of guthymol a few crystals of thymol and spatulate the mass thoroughly, when it will require a degree of pliability which renders its insertion in a tooth-cavity an operation of the simplest character.—Carlos Zacharias in *Cosmos.*

**Dignity of Prosthesis.** We are all coming back to prosthetic dentistry. It does not matter so much who does the work, you yourself, or the laboratory men, provided that it is properly done. Operative dentistry has in my opinion made the least progress, unless progress means the stuffing of a hole full of something. There has been no real effort to restore from an artistic standpoint the lost portion of a tooth.

The introduction of mechanical laboratories is a big mistake, but there will come a period when this branch will be put on a proper basis. Dentistry will be divided up. No one man can begin to digest and make use of all the good things that are offered to us.—Dr. Van Woert in *Items*.

**Non-Inflammable Celluloid.** "Zellir," according to the *Drogisten Zeitung*, is a celluloid acetate, which dissolves in acetic ester and camphor, giving plastic masses with the latter that can be rolled and moulded into various objects, just like celluloid.

Dr. Eichengruen, who has prepared this compound by acting upon cellulose with acetic acid, finds that it can also be worked up with non-inflammable substitutes of camphor, yielding a mass which possesses not only all the valuable properties of celluloid, but others as well. Thus it is possible, according to the manipulation, to obtain substances that are soft and flexible as leather and cloth, are elastic, as india rubber, but perfectly transparent, absolutely impervious to water, and non-inflammable.

**Nitrous Oxid the Anesthetic of Choice in Many Major Operations.** One of the most recent and authoritative advocates of nitrous oxid anesthesia in major surgery is Arthur Dean Bevan, M. D., of Chicago, who, in an address, as chairman of the Section on Surgery of the American Medical Association, reports that during the last three or four years he has used nitrous oxid and air as an anesthetic in a large number of cases, and that finding how easy it is to maintain satisfactory anesthesia for long periods, has gradually increased the range of its use until he now employs it in a large proportion of his surgical operations. Nitrous oxid is the anesthetic of choice in reducing fractures and dislocations, in opening abscesses and felons, in breaking up adhesions in joints, in draining empyemas and lung abscess, in exploratory laparotomies, in gall-bladder work, removing stones and drainage in kidney work, nephrotomy, and nephrolithotomy; in bladder work, suprapubic cystotomy for stone and in suprapubic prostatectomy.

**Temperaments and Occlusion of Artificial Teeth.** The Bonwill system of grinding and widening the space between the cusps of the bicuspids and molars in artificial teeth for the lateral movement of the lower jaw, is very essential for persons of certain temperaments. Those of sanguine and sub-sanguine temperaments rotate their jaw in masticating, the occlusion is only moderately firm, therefore it is necessary to have a free lateral movement of the jaw without the cuspids interfering. In the lymphatic and sub-lymphatic temperaments the occlusion is generally loose and flat with short cusps.

The Bonwill system of grinding and shaping the cusps for a free lateral movement of the jaw in the use of artificial teeth is very important for the sanguine, lymphatic and their sub-temperaments, especially the sanguine. Persons of nervous and sub-nervous, bilious and sub-bilious temperaments have but little lateral movement of the jaw in masticating; the occlusion is generally firm and close with long cusps.—G. North in *Summary.*

**Oral Prophylaxis.** Since the advent of bacteriology and the study of pathogenic micro-organisms, the medical profession throughout the world has been devoting more attention to the subject of oral prophylaxis, and I am one of those who believe that the reason why the field for the dentist has been so broad and the demand for his services so great is because science through the medium of the medical profession has taught the importance of oral prophylaxis. I do not believe it would be possible for our society to do a better work than to issue within as short a period of time as possible a monograph on this subject. The Odontological Society of Chicago made its reputation by issuing a little pamphlet on the treatment of pulpless teeth many years ago, and the time is now at hand when another monograph, most elaborately prepared, covering the whole field, should be issued. It would take the society a long time to prepare such a monograph which would be broad in every aspect, and this should be distributed broadcast, not only to the members of the dental, but to the members of the medical, profession, and in doing that we would impart to the people a knowledge of this important subject.—Dr. T. W. Brophy in *Review*.

**How Much Mercury.** To make a strong filling and one that will have close margins, get first the right proportion of mercury. Mix and work the material until the mass is perfectly smooth and plastic. Then it should take the finest skin markings of the fingers. Continue the kneading until the first slight evidence of stiffening becomes apparent and then make the filling quickly. It should stiffen at once on being pressed into the cavity, so that it will not draw back from the walls when the instrument is withdrawn, or move again when more material is pressed upon it. Do not mix a little in the mortar and then roll it a moment in the hand and place it in the cavity half or less than half amalgamated, because you will never have it in condition to make a good filling when mixed in that way. The filling will not shrink nor expand if the alloy is right. But it is not in any condition to make a filling that will ever become strong or good, and until we amend the mixing we will not have alloy fillings that are good. The working of an alloy, in getting the conditions and making good fillings, seems to be more difficult to learn than the making of a gold filling, but when it is once learned it is not so very difficult to do. But we must learn the manipulation just as we have to learn the manipulation of gold, and just as long as the body of the profession is careless about it we will have poor amalgam fillings.—Dr. G. V. Black in *Review*.

**Give Your Patients These Facts.**

1. Dental expense increases with neglect more rapidly than the interest on money.

2. The expense of dentistry is in proportion to the neglect of the patient.

3. The average pressure upon natural teeth is 150 pounds and only 25 pounds on plates.

4. Natural teeth are six times as serviceable as are artificial teeth.

5. With neglected teeth the greater part of the expense is *under* the fillings.

6. A good natural tooth though aching can be cured and do better service than a bridge.

7. Teeth may be deeply decayed and the patient not be aware of the cavities they contain.

8. Small fillings with strong surrounding walls save the teeth better than large fillings with frail surrounding walls.

9. Small fillings are less painful to insert than are the larger and deeper ones.

10. Small fillings take less time to insert than do the larger and deeper ones.

11. Dental expense is based in a measure upon the time reserved for the patient, therefore they should keep appointments promptly.

12. Decayed roots which cannot be crowned are injurious to the breath and health and should be extracted.

13. Health, beauty and comfort are dependent upon the proper care of the teeth.

14. If the natural teeth are put in good condition and attended to at frequent and regular intervals thereafter, they will render their owner many times over the service they have cost him.—H. W. McMillan in *Dental Review*.

